# Epigenetic Biomarkers as Predictors and Correlates of Symptom Improvement Following Psychotherapy in Combat Veterans with PTSD

**DOI:** 10.3389/fpsyt.2013.00118

**Published:** 2013-09-27

**Authors:** Rachel Yehuda, Nikolaos P. Daskalakis, Frank Desarnaud, Iouri Makotkine, Amy L. Lehrner, Erin Koch, Janine D. Flory, Joseph D. Buxbaum, Michael J. Meaney, Linda M. Bierer

**Affiliations:** ^1^Traumatic Stress Studies Division, Department of Psychiatry, Icahn School of Medicine at Mount Sinai, New York, NY, USA; ^2^Mental Health Care Center, PTSD Clinical Research Program and Laboratory of Clinical Neuroendocrinology and Neurochemistry, James J. Peters Veterans Affairs Medical Center, Bronx, NY, USA; ^3^Fishberg Department of Neuroscience, Icahn School of Medicine at Mount Sinai, New York, NY, USA; ^4^Laboratory of Molecular Neuropsychiatry, Department of Psychiatry, Icahn School of Medicine at Mount Sinai, New York, NY, USA; ^5^Department of Genetics and Genomics Sciences, Icahn School of Medicine at Mount Sinai, New York, NY, USA; ^6^Neuroscience Division, Douglas Mental Health University Institute, McGill University, Montreal, QC, Canada; ^7^The Agency for Science, Technology and Research, Singapore Institute for Clinical Sciences, Singapore

**Keywords:** PTSD, veterans, epigenetics, methylation, promoter, glucocorticoid receptor, FK506 binding protein 5, psychotherapy

## Abstract

Epigenetic alterations offer promise as diagnostic or prognostic markers, but it is not known whether these measures associate with, or predict, clinical state. These questions were addressed in a pilot study with combat veterans with PTSD to determine whether cytosine methylation in promoter regions of the glucocorticoid related *NR3C1* and *FKBP51* genes would predict or associate with treatment outcome. Veterans with PTSD received prolonged exposure (PE) psychotherapy, yielding responders (*n* = 8), defined by no longer meeting diagnostic criteria for PTSD, and non-responders (*n* = 8). Blood samples were obtained at pre-treatment, after 12 weeks of psychotherapy (post-treatment), and after a 3-month follow-up. Methylation was examined in DNA extracted from lymphocytes. Measures reflecting glucocorticoid receptor (GR) activity were also obtained (i.e., plasma and 24 h-urinary cortisol, plasma ACTH, lymphocyte lysozyme IC_50-DEX_, and plasma neuropeptide-Y). Methylation of the GR gene (*NR3C1)* exon 1F promoter assessed at pre-treatment predicted treatment outcome, but was not significantly altered in responders or non-responders at post-treatment or follow-up. In contrast, methylation of the FKBP5 gene (*FKBP51)* exon 1 promoter region did not predict treatment response, but decreased in association with recovery. In a subset, a corresponding group difference in FKBP5 gene expression was observed, with responders showing higher gene expression at post-treatment than non-responders. Endocrine markers were also associated with the epigenetic markers. These preliminary observations require replication and validation. However, the results support research indicating that some glucocorticoid related genes are subject to environmental regulation throughout life. Moreover, psychotherapy constitutes a form of “environmental regulation” that may alter epigenetic state. Finally, the results further suggest that different genes may be associated with prognosis and symptom state, respectively.

## Introduction

Cytosine methylation of glucocorticoid related genes represents an epigenetic modification thought to underlie the developmental programing of hypothalamic-pituitary-adrenal (HPA) axis function (1). Plasticity of the epigenome appears to constitute a molecular mechanism whereby genetic predispositions may be influenced by environmental exposures resulting in sustained alterations in gene expression and protein synthesis (2–4). Epigenetic modifications of a glucocorticoid receptor (GR) gene promoter were first described in the rat as a mechanism by which variations in parent – offspring interactions influence HPA-axis and behavioral responses to stress (5, 6). Maternal care regulates the methylation state of the GR exon 1_7_ promoter in hippocampus, which in turn, regulates GR expression, the capacity for glucocorticoid negative feedback, and HPA-axis responses to stress (5, 7). Subsequent studies in humans showed that childhood adversity associates with higher methylation of the GR exon 1F promoter (the human ortholog of the rat exon 1_7_ promoter sequence) lower hippocampal GR expression and increased HPA-axis responses to stress (8, 9).

Recent studies reveal additional mechanisms for the influence of childhood adversity on GR signaling and HPA-axis function. FK506 binding protein 5 (FKBP5) regulates intracellular GR signaling by decreasing ligand binding and restricting GR translocation to the nucleus (10, 11). GR activation induces *FKBP51* (the FKBP5 gene) transcription, thus establishing an intracellular feedback loop that moderates GR sensitivity (12). FKBP5 genetic variants in interaction with childhood adversity predict the risk for affective disorders, including major depression, suicide attempts, and PTSD (13–16). Moreover, the methylation state of selected CpGs across the *FKBP51* gene is determined by an interaction between sequence-polymorphism and childhood adversity, and modulates sensitivity of FKBP5 to GR regulation (17). Various aspects of the GR (*NR3*C1) and FKBP5 genes, including genotype and gene expression, have been implicated in PTSD (12, 13, 15, 18–27). Low FKBP5 gene expression in PTSD has been associated with low plasma cortisol and PTSD severity (21, 24). Taken together, these findings suggest that childhood adversity influences the epigenetic state and transcriptional activity of genes that regulate HPA-axis responses to stress.

Importantly, stress reactivity predicts the risk for multiple affective disorders, as well as PTSD (28). Early adverse experiences are risk factors for PTSD following adult trauma exposures (29–31); thus the associated epigenetic states may represent a molecular mechanism responsible for altering subsequent responses to environmental adversity (4, 32). Neuroendocrine studies reveal that the development of PTSD following trauma exposure is associated with pre-traumatic biological markers that reflect prior sensitization to stress (33). Relatively stable changes in methylation potentially explain the chronicity and tenacity of symptoms observed in PTSD. In PTSD there is neither a complete restoration of baseline hormone levels following trauma, nor do persons with this condition feel that they have returned to a pre-trauma psychological state. PTSD is a condition that has been associated with low glucocorticoid levels, enhanced GR sensitivity, and insufficient glucocorticoid signaling (34–37). Epigenetic signals associated with childhood adversity offer a potential explanation both for why stress responses do not abate once an immediate threat is no longer present, as in the case of PTSD, and for the fact that some persons are at greater risk than others for the development of PTSD (32, 38). In fact, many of the alterations noted in PTSD have been demonstrated in association with early adversity regardless of the subsequent development of PTSD in adults (9, 17, 39, 40). On the other hand, persons who develop PTSD can also recover from this condition either spontaneously or in response to treatment (41). Moreover, an emerging trajectory in PTSD is one in which there are fluctuating symptoms, which maybe mediated by external post-traumatic environmental circumstances. This raises the possibility that some epigenetic changes, originally induced by the environment, change over time in response to subsequent challenges.

The goal of the current study was to examine methylation of the GR and FKBP5 genes – and associated downstream neuroendocrine measures, cortisol, and NPY, before and after prolonged exposure (PE) psychotherapy in veterans with PTSD. The exon 1F promoter was selected as the most biologically relevant GR promoter region for methylation analysis because this region corresponds to exon 1_7_ of the rat GR gene, shown to be differentially methylated in the rat hippocampus based on variations in maternal care (5), and in human peripheral blood and hippocampal post-mortem tissue in association with child abuse (9, 39, 40). We hypothesized that higher GR exon 1F promoter methylation would predict treatment response and “normalization” of PTSD related biology at post-treatment time-points but would not itself change appreciably over time. We also examined the FKBP5 exon 1 promoter methylation and, based on previously observed changes in FKBP5 gene expression in association with PTSD symptom severity (24), we hypothesized that *FKBP5* promoter methylation would change in responders, in association with glucocorticoid related measures.

The examination of biological measures in association with PTSD symptom change following an efficacious psychotherapy trial was designed to yield a sample with a variable degree of symptom improvement, with some showing large decreases in symptom severity, and others, minimal or moderate change. An additional advantage of this approach is the ability to modify symptoms without introducing exogenous medications that might have direct effects on the biological measures of interest. The participants for this study were drawn from a larger pool of combat veterans that were examined as part of an effort to identify neuroendocrine markers (e.g., cortisol, NPY) that would distinguish diagnostic, state related, and recovery markers in combat veterans randomized to PE or a minimal attention (MA) condition. To accomplish the larger objective, combat veterans were assessed for blood and urinary biomarkers prior to, and after completing, 12 weeks of treatment – either PE or MA – and after a 3-month naturalistic follow-up (for those who received PE). The direct manipulation of target symptoms with psychotherapy within a relatively short period of time (weeks to months) permits identification of biomarkers associated with relatively rapid symptom change and treatment-associated recovery. Assessment prior to and following psychotherapy allows differentiation of prognostic indicators from state markers of symptom change. Markers that do not change as symptoms improve may be prognostic indicators or reflect measures associated with risk for PTSD. Previous results from a preliminary study of combat veterans demonstrated that GR responsiveness predicted treatment outcome (42). Because it was of interest to draw specific conclusions about symptom change in association with a structured psychotherapy, in this report we only include participants in the active arm (i.e., who received and completed PE) who completed the pre-treatment, post-treatment, and follow-up assessments.

## Materials and Methods

### Participants

This report represents a subsample (*n* = 16) of a larger study of 113 combat veterans who enrolled in a clinical trial comparing the effects of PE to a MA condition, conducted at the James J. Peters Bronx VA Medical Center (JJP BVAMC). Results of the subset of completers will be reported elsewhere. The current subsample comprised 14 men and 2 women who completed PE treatment. Nine were Vietnam veterans, and seven had recently returned from active duty in Iraq or Afghanistan. The decision to study PE completers in this subset was based on two considerations. First, following MA, the participants were allowed to begin active psychotherapy. For this reason, the initial study did not have a follow-up evaluation for those receiving MA. Second, by comparing participants who received the same intervention, biological correlates of symptom severity are not confounded with effects of treatment type.

As the molecular measures reported here were not part of the original protocol, selection of this subgroup was based on (1) having agreed to the future use of their biological samples; (2) having completed all three evaluations (pre-treatment, post-treatment, and follow-up); (3) having participated in the PE condition; and (4) having sufficient remaining sample for the analysis of promoter methylation of GR and FKBP5 genes after other study measures had been obtained. Participants in this subsample were not appreciably different from those who completed PE in the parent study with respect to pre-treatment demographic or clinical variables, or post-treatment measures. All procedures were approved by the IRB at the JJP BVAMC, all participants signed written, informed consent prior to initiation of study procedures.

#### Inclusion/exclusion criteria

Following a comprehensive medical (including lab testing) and psychological evaluation, participants were excluded if they did not experience a Criterion A traumatic event during military service or meet DSM-IV criteria for current PTSD with a duration of at least 6 months. Additional exclusion criteria included having significant illness that would interfere with interpretation of biological data, such as insulin-dependent diabetes, seizure disorder, or any disease requiring ongoing treatment with systemic steroids; regular use of benzodiazepines or oral steroids; a BMI >40; smoking more than two packs per day; meeting criteria for substance abuse or dependence within the last 6 months; a lifetime history of schizophrenia, schizoaffective disorder, bipolar disorder, obsessive compulsive disorder, or being in any acute clinical state that necessitated prompt initiation of pharmacotherapy or other treatment, including assessed suicide risk. Veterans receiving psychotropic medications for PTSD were eligible to participate if they had maintained a stabilized therapeutic dose for a minimum of 2 months prior to randomization.

### Procedure

A comprehensive psychological evaluation was performed by a clinical psychologist at the three study time-points (pre- and post-treatment, follow-up). Several structured diagnostic instruments were used including the Structured Clinical Interview for DSM-IV (SCID) (43), and the Clinician Administered PTSD Scale (CAPS) (44). The CAPS additionally provided a continuous measure of symptom severity of PTSD. The PTSD Symptom Scale – Self-Report Version (PSS-SR) was used as a self-report of PTSD symptoms (45). Two self-report measures were administered to assess childhood trauma and life events. The Childhood Trauma Questionnaire (CTQ) was used to assess early trauma (46), and the Deployment Risk and Resiliency Inventory (DRRI) to access military and civilian life events pre- and post-deployment (47). For all subjects, an independent evaluator (i.e., not the individual who provided treatment) assessed clinical outcome following treatment.

#### Biological measures

The primary molecular measures included GR- and FKBP5-promoter methylation. These were obtained in parallel with the psychological assessments. We also examined FKBP5 gene expression in subjects for whom there was sufficient sample. A battery of HPA-axis markers was examined as part of the parent study to assess basal cortisol levels and GR responsiveness. Reported here are the biological measures that should be functionally related to the molecular measures and/or may vary in response to symptom change. These include basal plasma cortisol, 24 h-urinary cortisol levels, plasma ACTH, and cortisol responses to a low dose (0.50 mg) dexamethasone suppression test (DST), glucocorticoid sensitivity as assessed by the lymphocyte lysozyme IC_50-DEX_, and plasma NPY.

#### Sample processing and hormone determination

Blood samples pre- and post-dexamethasone were collected as previously described (48). Plasma was extracted from EDTA containing tubes, aliquoted, and frozen at −80°C until subsequent hormonal analysis. Urine samples were collected over a 24-h period as previously described (49). Cortisol (plasma and urinary), dexamethasone, and NPY were determined by radioimmunoassay as previously described (48, 50). Plasma ACTH was determined using an enzyme-linked immunosorbent assay (ELISA; ALPCO Diagnostics, Salem, NH, USA). The intra- and inter-assay coefficients of variation were 4.7 and 7.1% for ACTH, 2.3 and 6.1% for cortisol, 8.0 and 9.0% for dexamethasone, and 3.5 and 11.6% for NPY, respectively.

#### Peripheral blood mononuclear cells isolation

Peripheral blood mononuclear cells (PBMCs) were purified from basal EDTA pretreated blood by Ficoll-Paque (Amersham, UK) using Accuspin tubes (Sigma-Aldrich, Saint Louis, MO, USA). After two washes in Hanks’ Balanced Salt Solution (Life Technologies, Grand Island, NY, USA), PBMCs were counted with a hemocytometer. Some cell pellets were immediately used for determination of lysozyme IC_50-DEX_ as previously described (51). Some cell pellets were quickly frozen, stored at −80°C, and later used for DNA extraction (see below) and a portion of cell pellets was dissolved in TRIzol Reagent (Invitrogen, CA, USA) by adding 1 ml of the reagent per 1 × 10^7^ cells, quickly frozen, stored at −80°C, and later used for RNA extraction (see below).

#### DNA cytosine methylation sodium bisulfite mapping

**Figure 1:**
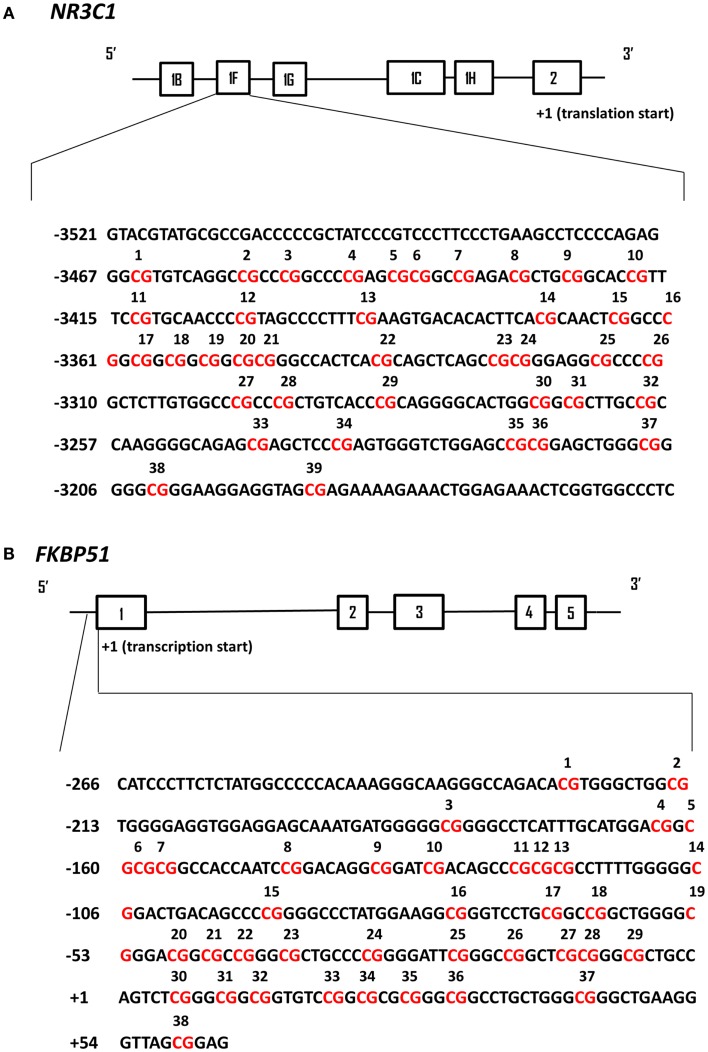
**Schematic representation of human *NR3C1* and *FKBP51* exon 1 promoter regions analyzed by DNA cytosine methylation bisulfite mapping**. In both panels the solid black line boxes with a number represent the different exons and the 5′–3′ orientation goes from left to right. **(A)** The *NR3C1* gene 5′ region is composed of multiple first exons and the translation start site is located within exon 2. The numbering of exon 1F promoter is based on the translational start site (+1). The CpG sites that have been analyzed by bisulfite sequencing are in red and numbered. **(B)** The *FKBP51* gene proximal promoter region is numbered based on the transcriptional start site (+1) of exon 1. The CpG sites that have been analyzed by bisulfite sequencing are in red and numbered.

Genomic DNA was extracted from frozen PBMC pellets following the Flexigene DNA kit protocol (Qiagen, Valencia, CA, USA). Methylation mapping of the human GR exon 1F promoter (Figure 1A) was performed following Dr. Meaney’s laboratory recommendations and as previously described for human hippocampus (9). The methylation mapping method for the human FKBP5 proximal promoter located upstream of exon 1 (Figure 1B) was developed in Dr. Yehuda’s laboratory. Sodium bisulfite treatment was carried out according to the EpiTect Bisulfite kit protocol (Qiagen, Valencia, CA, USA). In each sodium bisulfite conversion reaction, 0.8 μg of genomic DNA was used. In the same experiment, 0.8 μg of Universal Methylated Standard (Zymo Research, Irvine, CA, USA) was treated with sodium bisulfite to check completion of the sodium bisulfite reaction. The genomic region of the human GR exon 1F promoter was subjected to PCR amplification using the following primer sequences: 5′-GTG GTG GGGGAT TTG-3′ (forward); 5′-ACCTAATCTCTCTAAAAC-3′ (reverse) following previously published procedures (9). The thermocycler protocol involved an initial denaturation (5 min, 95°C), 35 cycles of denaturation (1 min, 95°C), annealing (2 min 30 s, 55°C), and extension (1 min, 72°C), and then a final extension (5 min, 72°C) with subsequent cooling at 4°C. The resulting PCR product was subjected to another round of PCR, using the following nested primers: 5′-TTTTTGAAGTTTTTTTAGAGGG-3′ (forward); 5′-AATTTCTCCAATTTCTTTTCTC-3′ (reverse). The thermocycler protocol was the same as the initial PCR procedure except that the extension step was prolonged to 10 min. The genomic region of the human FKBP5 exon 1 promoter was subjected to PCR amplification using the following primer sequences: 5′-GGTAGGTTTTGTGGATAGATAGGA-3′ (forward); 5′-ACTCCGCTAACCCTTCAAC-3′ (reverse). The thermocycler protocol involved an initial denaturation (4 min, 95°C), 35 cycles of denaturation (30 s, 95°C), annealing (30 s, 45°C), and extension (1 min, 72°C), and then a final extension (10 min, 72°C) with subsequent cooling at 4°C. The resulting PCR product was subjected to another round of PCR, using the following nested primers: 5′-AGGGGGTGTTAGTTTTTATTATTTTTT-3′ (forward); 5′-ACTCCGCTAACCCTTCAAC-3′ (reverse). The thermocycler protocol was the same as the initial PCR procedure. The resulting PCR products were analyzed on a 2% agarose gel and then purified using QIAquick PCR purification kit (Qiagen, Valencia, CA, USA). The PCR products were subcloned using a PCR product cloning kit (Qiagen) and individual plasmid containing the ligated promoter regions were extracted and sequenced (Genewiz, Inc., South Plainfield, NJ, USA). The sequences for 20 individual clones were aligned and analyzed in the DNA Alignment software program BioEdit (Ibis Biosciences, Carlsbad, CA, USA). The DNA samples were analyzed in batches of 20–30 samples. Variability in the DNA bisulfite treatment did not exceed 2% between the batches.

#### Gene expression

RNA, from Trizol-dissolved PBMCs, was extracted using commercially available kits (RNeasy Mini Kit and RNeasy MinElute Cleanup Kit, Qiagen). Extracted RNA was evaluated for its quality using NanoDrop 2000 Spectrophotometer (Thermo Scientific). RNA was aliquoted and stored at −80°C until use.

For GR exon 1F expression, cDNA synthesis was completed using Maxima reverse transcriptase (Thermoscientific) and GR target oligo (CAG GGG TGC AGA GTT CGA TG) since GR expression levels are very low in blood cells. Quantitative real-time PCR was performed with a LightCycler 480 (Roche Applied Science). *NR3C1* exon 1F primers (forward primer 5′-AAG AAA CTG GAG AAA CTC GGT GGC-3′, reverse primer 5′-TGA GGG TGA AGA CGC AGA AAC CTT-3′) and RT^2^ PCR primer sets for two endogenous reference genes (β2 microglobulin, Catalog no. PPH01094E; SABiosciences and glyceraldehyde-3-phosphate dehydrogenase, Cat#PPH14985F, Sabioscience) were used. Only one cDNA was amplified in each PCR (monoplex).

For FKBP5 expression, cDNA was synthesized by reverse transcription reaction using High capacity cDNA Archive Kit (Applied Biosystems). Real-time PCR was performed using an ABI Step One Plus Real-Time PCR Instrument (Applied Biosystems) and TaqMan probes (Applied Biosystems). The primers used to target exon junctions 7–8 and 8–9 of the *FKBP51* gene, and four endogenous reference genes, have been previously described (21). Only one cDNA was amplified in each PCR (monoplex).

The reactions were run in triplicate for each sample and were quantitated by selecting the amplification cycle when the PCR product of interest was first detected (threshold cycle, Ct). To account for the differences in the amounts of input material across samples, the expression level of each transcript in each sample was normalized to the geometric mean of the expression levels of the endogenous reference genes using the 2^−ΔΔCt^ method.

### Statistical analysis

Responder status was defined by the presence or absence of PTSD at post-treatment evaluation, as determined by an independent psychologist using the CAPS for DSM-IV. For GR and FKBP5, number of methylated sites in the CpG region examined for each subject was calculated by observing the percentage of methylated clones at each site and then totaling the number of sites with percentages greater than zero. Because the number of individual sites examined was 39 for GR and 38 for FKPB5, the potential range of number of methylated sites for GR is 0–39, with an actual range of 1–16; the potential range for FKBP5 is 0–38, with an actual range of 2–20. An alternative measure for promoter methylation was the sum % methylation. For this measure, at each site of the promoter region, the total number of methylated clones (out of 30) was converted to a percentage. The percentages across all sites were then added to create a total summed percentage of methylation.

Measures of central tendency and variability (mean and SE) were calculated at baseline, treatment completion, and follow-up for all continuous primary and secondary clinical outcome measures and biological variables. Baseline comparisons of group differences were conducted using independent samples *t*-tests for continuous variables and chi-square analysis for categorical variables. Correlation analyses were conducted to determine appropriate covariates for repeated measures analysis. Repeated measures ANOVAs and ANCOVAs were conducted using responders and non-responders to explore within and between group changes on biological and psychological measures in order to determine predictors and correlates of treatment outcome. Additional bivariate correlations were used to measure association of GR- and FKBP5-promoter methylation at pre-treatment with clinical and other biological variables at post-treatment or follow-up and of post-treatment variables with those at follow-up. For the correlational analysis, the number of methylated sites was selected as the most sensitive measure of methylation in this study. Statistical significance for all analyses was set at *p* < 0.05.

## Results

### Demographic, descriptive, and clinical measures

Table 1 reports comparisons of the responder and non-responder groups at baseline on a variety of demographic and descriptive characteristics. There were significant group differences in age indicating that responders tended to be younger, had PTSD for a shorter duration, and had fewer total lifetime traumatic events, than non-responders. A chi-square analysis of the number of veterans in the two conflicts in relation to responder status did not reach statistical significance in this small sample. Moreover, initial PTSD symptom severity was comparable for responders and non-responders, as assessed by clinician or self-report (Table 2).

**Table 1:** **Baseline characteristics comparing responders to non-responders**.

	Responders (*n* = 8)	Non-responders (*n* = 8)		
	M (SD) or %	M (SD) or %	
Age	41.25 (17.82)	57.88 (7.45)	*t*_(9.4)_^d^ = *−*2.435	***p* ***=*** 0.037**
Years of education	13.38 (2.20)	15.50 (2.39)	*t*_(14)_ = *−*1.850	ns
Marital status	Single (37.5%)	Single (37.5%)	χ^2^_(1)_ = 1.000	ns
	Married or living with partner (62.5%)	Married or living with partner (62.5%)	
Ethnicity	Hispanic (37.5%)	Hispanic (50%)	χ^2^_(2)_ = 0.476	ns
	Black (37.5%)	Black (37.5%)	
	White (25%)	White (12.5%)	
Conflict	OEF/OIF (62.5%)	OEF/OIF (25%)	χ^2^_(1)_ = 2.286	ns
	Vietnam (37.5%)	Vietnam (75%)	
Stabilized on psychotropics	Yes (62.5%)	Yes (62.5%)	χ^2^_(1)_ = 1.000	ns
Lifetime CAPS^a^ total score	92.75 (12.70)	105.25 (15.15)	*t*_(14)_ = *−*1.789	ns
CTQ^b^ total	9.43 (3.72)	10.81 (4.02)	*t*_(14)_ = *−*0.717	ns
Time since first DRRI^c^ trauma (years)	25.00 (17.85)	45.00 (8.83)	*t*_(10.2)_^d^ = 2.841	***p* = 0.017**
DRRI pre-deployment life events	4.50 (3.16)	7.25 (3.15)	*t*_(14)_ = *−*1.742	ns
DRRI post-deployment life events	5.13 (3.68)	10.38 (2.56)	*t*_(14)_ = *−*3.312	***p* ***=*** 0.005**
DRRI total life events	9.63 (5.80)	17.63 (3.16)	*t*_(14)_ = *−*3.424	***p* ***=*** 0.004**

^a^ Clinician Administered PTSD Scale;

^b^ Childhood Trauma Questionnaire;

^c^ Deployment Risk and Resilience Inventory;

^d^ unequal variance *t*-test.

**Table 2:** **Interview and self-report measures at before and after treatment and at 12-week follow-up in Responders (R) and Non-responders (NR)**.

	Pre-treatment		Post-treatment		Follow-up		*p*-Value		
	R	NR	R	NR	R	NR	Group	Time	G ***×*** T
Current PTSD severity^a^	75.00 (6.39)	81.75 (6.39)	26.50 (6.78)	70.50 (6.78)	30.00 (7.72)	61.75 (7.72)	**0.004**	**0.000**	**0.003**
Self-rated PTSD severity^b^	36.50 (2.39)	35.17 (2.76)	18.75 (3.98)	34.50 (4.60)	14.63 (3.15)	32.00 (3.63)	**0.016**	**0.000**	**0.004**

^a^ Clinician Administered PTSD Scale (CAPS);

^b^ PTSD Symptom Scale-Self-Report (PSS-SR).

### Treatment response and clinical indicators

Table 2 also describes changes in measures of PTSD. Consistent with defining groups on the basis of their diagnostic status at post-treatment, there were significant group × time interactions for PTSD symptom severity.

### Methylation of a GR promoter

**Figure 2:**
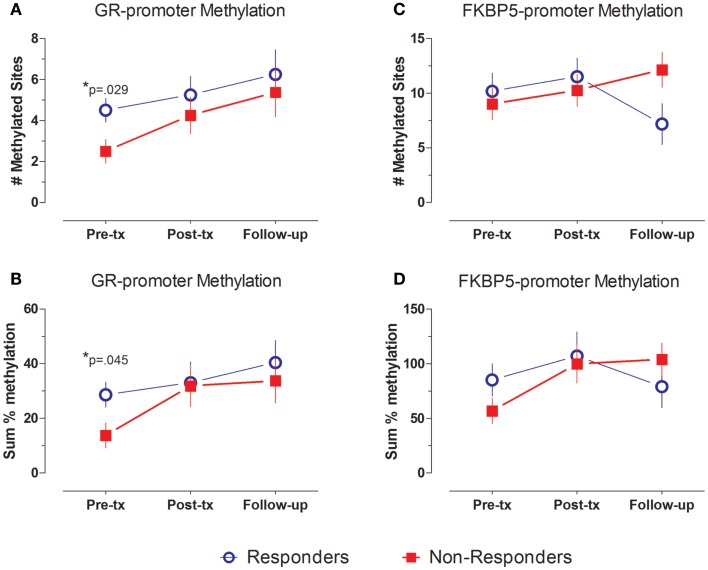
**GR- and FKBP5-promoter methylation at pre-treatment, post-treatment and follow-up**. GR- **(A,B)** and FKBP5-promoter **(C,D)** methylation shown by mean ± SE of number of methylated sites **(A,C)** or sum % methylation **(B,D)**. Responders (*n* = 8) to treatment are represented by blue, open circles and non-responders (*n* = 8) by red squares. For GR exon 1F promoter methylation, there is a significant group difference at Pre-treatment, but no main effect of time and no group by time interaction. FKBP5 promoter methylation shows a significant group by time interaction, but no main effects of group or time. Statistical significance was set at *p* < 0.05.

Levels of methylation across the GR exon 1F promoter were generally low, as expected of CpG sites lying within a CpG island. Figure 2A demonstrates a significant group difference in the number of CpG methylated sites across the GR exon 1F promoter between responders and non-responders at pre-treatment (*t*_14_ = 2.43, *p* = 0.029), with a greater average number of methylated sites in responders (4.5 ± 0.6) than non-responders (2.5 ± 0.6). A similar pre-treatment difference in GR exon 1F promoter methylation was observed when the sum % methylation measure (Figure 2B) was used (*t*_14_ = 2.29, *p* = 0.045; responders 28.7 ± 5.9, non-responders: 13.8 ± 2.8).

In repeated measures analysis of number of methylated sites, there was a main effect of group (*F*_1,14_ = 7.584, *p* = 0.016) across the three time-points, but no significant effect of time (*F*_2,28_ = 2.41, ns) or group × time interaction (*F*_2,28_ = 0.171, ns). The significant group effect reflects higher level of number of methylated CpG sites in samples from responders compared to non-responders. Similar effects at a trend level of significance were observed when using the sum % methylation measure (group: *F*_1,14_ = 3.627, *p* = 0.078, trend; time: *F*_2,28_ = 2.22, ns, group × time interaction: *F*_2,28_ = 0.401, ns).

**Figure 3:**
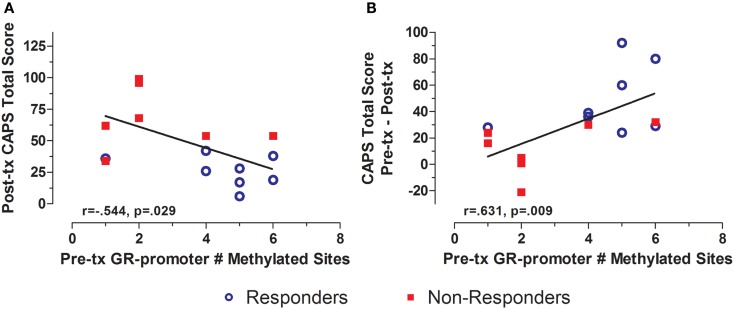
**Relationship between GR exon 1F promoter methylation at pre-treatment and PTSD symptom severity at post-treatment**. Correlations of pre-treatment GR exon 1F promoter methylation (# of methylated sites) with post-treatment CAPS total score **(A)** and change in CAPS total score from pre- to post-treatment **(B)**. Responders (*n* = 8) to treatment are represented by blue, open circles and non-responders (*n* = 8) by red squares. The higher number of GR exon 1F promoter methylated sites at pre-treatment corresponded to a lower CAPS total score at Post-treatment and a greater reduction in symptoms from pre- to post-treatment. Correlation coefficients are denoted in the different panels. Statistical significance was set at *p* < 0.05.

**Figure 4:**
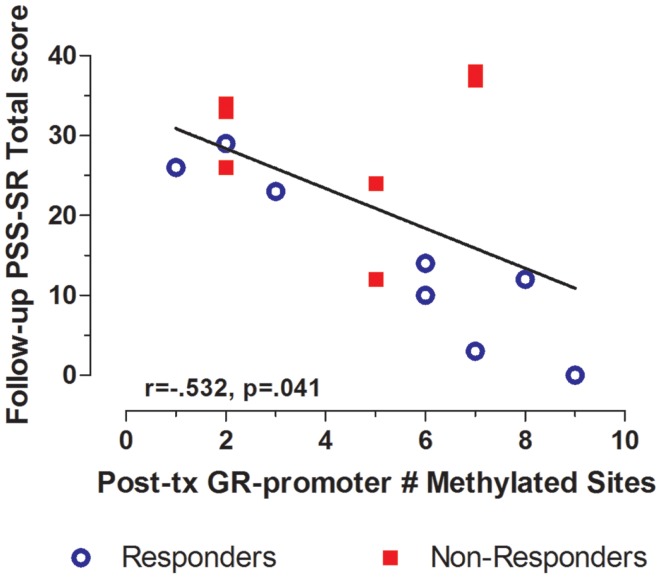
**Relationship between GR exon 1F promoter methylation at post-treatment and self-reported PTSD symptom severity at follow-up**. Correlations of Post-treatment GR exon 1F promoter methylation (# of methylated sites) with PSS-SR total score at follow-up. Responders (*n* = 8) to treatment are represented by blue, open circles and non-responders (*n* = 7) by red squares. The higher number of GR exon 1F promoter methylated sites at post-treatment corresponded to a lower PSS-SR total score at follow-up (*r* = −0.532, *p* = 0.041). Statistical significance was set at*p* < 0.05.

Treatment response for individual subjects was predicted by pre-treatment GR exon 1F promoter methylation. Pre-treatment levels of GR exon 1F promoter methylation were significantly correlated with both post-treatment PTSD symptom severity (Figure 3A) and the change in symptom severity from pre- to post-treatment (Figure 3B). Higher post-treatment GR exon 1F promoter methylation also predicted lower self-reported (but not clinician-rated) PTSD symptoms at follow-up (Figure 4).

**Figure 5:**
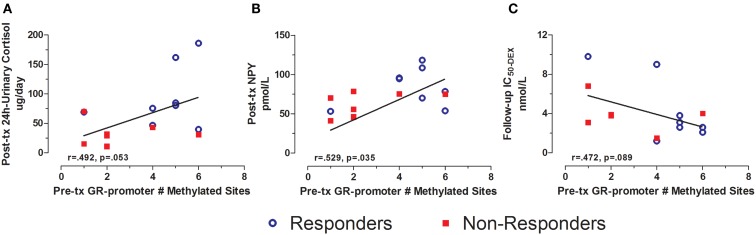
**Relationship between GR exon 1F promoter methylation at pre-treatment and urinary cortisol, NPY, and IC50-DEX at post-treatment or follow-up**. Correlations of pre-treatment GR exon 1F promoter methylation (# of methylated sites) with adjusted 24 h-urinary cortisol (see below) at post-treatment **(A)**, neuropeptide-Y (NPY) at post-treatment **(B)**, and IC_50-DEX_ at follow-up **(C)**. Responders (*n* = 8) to treatment are represented by blue, open circles and non-responders (*n* = 8, *n* = 6 for IC_50-DEX_) by red squares. The higher number of GR exon 1F promoter methylated sites at Pre-treatment corresponded to higher adjusted 24 h-urinary cortisol and NPY at Post-treatment and lower IC_50-DEX_ at follow-up (trend). About 24 h-urinary cortisol was adjusted for BMI and gender using linear regression and adding unstandardized residuals to the initial raw levels. Correlation coefficients are denoted in the different panels. Statistical significance was set at *p* < 0.05.

Pre-treatment GR exon 1F promoter methylation additionally predicted several post-treatment biological measures. Pre-treatment GR exon 1F promoter methylation was positively associated with post-treatment 24 h-urinary cortisol levels (Figure 5A) and plasma NPY (Figure 5B). Although only at a trend level of significance, pre-treatment GR exon 1F promoter methylation was associated with follow-up glucocorticoid sensitivity as determined by the lymphocyte lysozyme test 3 months after treatment ended (Figure 5C). Note that lower IC_50-DEX_ indicates greater glucocorticoid sensitivity. Importantly, there were no significant correlations observed cross-sectionally between GR exon 1F promoter methylation and PTSD symptoms or other endocrine measures in this sample.

### Methylation of the FKBP5 promoter

In contrast to the findings for the GR exon 1F promoter, FKBP5 promoter number of methylated sites showed variation in association with treatment outcome reflected in a significant group by time interaction effect (*F*_2,24_ = 4.576, *p* = 0.021). Responders showed a decrease, whereas non-responders showed an increase in FKBP5 promoter methylation over this same period (Figure 2C). This interaction effect was confirmed at a trend level of significance using the sum % methylation measure (*F*_2,22_ = 4.276, *p* = 0.063, trend; Figure 2D) and was likely due to decreased levels of FKBP5 promoter methylation among responders from post-treatment to the follow-up time-point.

**Figure 6:**
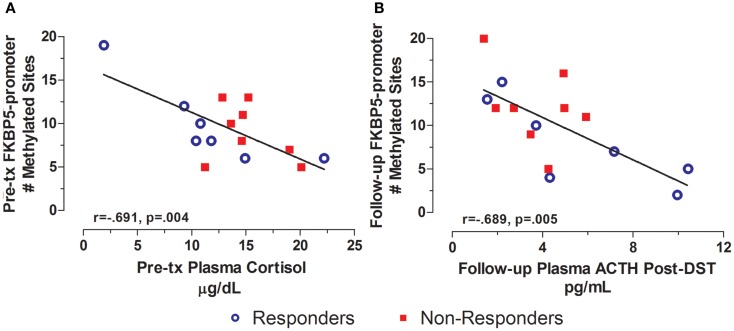
**Cross-sectional relationship between FKBP5 promoter methylation and HPA-axis endocrine markers**. Correlations of pre-treatment plasma cortisol with pre-treatment FKBP5 promoter methylation (# of methylated sites) **(A)** and follow-up adjusted post low dose dexamethasone suppression test (DST) plasma ACTH with follow-up FKBP5 promoter methylation (# of methylated sites) **(B)**. Responders (*n* = 7) to treatment are represented by blue, open circles and non-responders (*n* = 8) by red squares. Higher pre-treatment plasma cortisol and Follow-up post-DEX adjusted ACTH associated with lower FKBP5 promoter methylation at pre-treatment and Follow-up, respectively. Follow-up post-DST ACTH was adjusted for dexamethasone levels and pre-DST ACTH levels using linear regression and unstandardized residuals were added to the initial raw levels. Correlation coefficients are denoted in the different panels. Statistical significance was set at *p* < 0.05.

In contrast to GR exon 1F promoter methylation, for which measures at pre-treatment predicted symptoms or biological measures at subsequent time-points, FKBP5 promoter methylation tended to associate cross-sectionally with biological measures at pre- and post-treatment time-points. For example, FKBP5 promoter methylation at pre-treatment was significantly correlated with plasma cortisol levels (Figure 6A) such that higher FKBP5 promoter methylation was correlated with lower cortisol levels at pre-treatment, a result compatible with our previous findings of lower FKBP5 gene expression in PTSD (21). Following treatment, FKBP5 promoter methylation was significantly negatively correlated with pituitary response to dexamethasone as measured by ACTH levels following the administration of low dose dexamethasone (Figure 6B). A similar correlation was observed at a trend level of significance with post-dexamethasone cortisol (*r* = −0.509, *n* = 15, *p* = 0.053). Since lower ACTH or cortisol levels following the low dose DST reflect a greater negative-feedback inhibition of the HPA-axis, these findings suggest that greater GR responsiveness associated with higher levels of FKBP5 promoter methylation.

**Figure 7:**
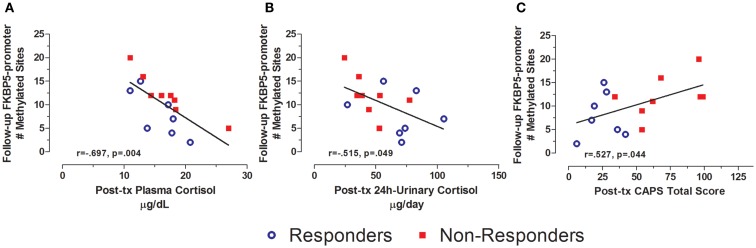
**Relationship between HPA-axis endocrine markers and PTSD severity at post-treatment and FKBP5 promoter methylation at follow-up**. Correlations of post-treatment plasma cortisol **(A)**, post-treatment 24-h urinary cortisol **(B)**, and post-treatment CAPS total score **(C)** with Follow-up FKBP5 promoter methylation (# of methylated sites). Responders (*n* = 7) to treatment are represented by blue, open circles and non-responders (*n* = 8) by red squares. Both higher plasma cortisol and higher 24 h-urinary cortisol associated with lower FKBP5 promoter methylation at follow-up. Higher post-treatment CAPS total scores associated with more FKBP5 promoter methylation at follow-up. Correlation coefficients are denoted in the different panels. Statistical significance was set at *p* < 0.05.

Levels of FKBP5 promoter methylation at follow-up were also associated with measures of both endocrine function and symptoms at post-treatment (Figure 7). Thus, FKBP5 promoter methylation at follow-up was significantly correlated with both plasma cortisol and 24 h-urinary cortisol at post-treatment (Figures 7A,B, respectively), suggesting that FKBP5 promoter methylation may be associated with changes in HPA-axis activity, in association with changes in symptom expression, rather than reflecting upstream regulation of cortisol. The finding that post-treatment PTSD severity was correlated with FKBP5 promoter methylation at follow-up (Figure 7C) is consistent with this idea.

### Expression of GR exon 1F and FKBP5

Because the assays for gene expression were not planned at the outset of this clinical trial, biological material was only available at the follow-up time-point and not for all subjects. Treatment responders showed higher expression of the GR exon 1F and FKBP5 genes compared with non-responders (for GR exon 1F: 1.0 ± 0.1 and 0.4 ± 0.1, respectively, for FKBP5 exon 8/9: 3.1 ± 1.4 and 1.2 ± 0.4, respectively). This difference was significant only for GR exon 1F (*t*_4_ = 2.29, *p* = 0.019) in this small sample.

Plasma cortisol was positively correlated with FKBP5 gene expression (for exon 7/8 transcript: *r* = 0.654, *n* = 10, *p* = 0.040) and negatively correlated with GR exon 1F expression (*r* = −0.853, *n* = 6, *p* = 0.031) at follow-up. FKBP5 gene expression also negatively correlated with the decline in cortisol in response to dexamethasone (for the exon 7/8 transcript: *r* = −0.869, *n* = 10, *p* = 0.002).

Endocrine markers assessed at pre- and post-treatment correlated with FKBP5 gene expression at follow-up. For example, ACTH levels following dexamethasone at pre-and post-treatment predicted lower GR exon 1F, and higher FKBP5 gene expression at the 7/8 transcript (at pre-treatment, *r* = −0.929, *n* = 6, *p* = 0.022 for GR exon 1F and *r* = 0.712, *n* = 9, *p* = 0.031 for the FKBP5 exon 7/8 transcript; at post-treatment *r* = −0.616, *n* = 6, ns for GR exon 1F and *r* = 0.768, *n* = 10, *p* = 0.016 for the FKBP5 exon 8/9 transcript). This suggests that those showing relatively lower pituitary GR responsiveness before and/or after treatment were most likely to demonstrate a treatment (or symptom) induced decrease in GR gene expression and/or increase in FKBP5 gene expression, a likely consequence of demethylation of the FKBP5 promoter region described above.

### Methylation and traumatic life events

**Figure 8:**
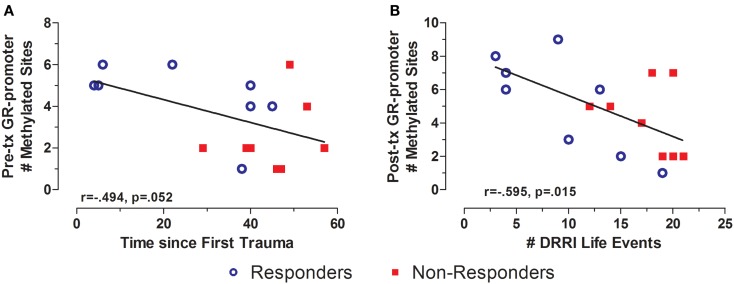
**Relationship between significant and potentially traumatic events at pre-treatment and GR exon 1F promoter methylation**. Correlations of time since first trauma assessed on the Deployment Risk and Resiliency Inventory (DRRI) with the with Pre-treatment GR exon 1F promoter methylation **(A)** and number of life events as assessed on the DRRI with GR exon 1F promoter methylation at post-treatment **(B)**. Responders (*n* = 8) to treatment are represented by blue, open circles and non-responders (*n* = 8) by red squares. Longer time since first trauma and greater number of traumatic life events both associated with lower GR exon 1F promoter methylation, at pre-treatment and post-treatment, respectively. Correlation coefficients are denoted in the different panels. Statistical significance was set at *p* < 0.05.

There was a significant difference in total life events in responders vs. non-responders (Table 1). GR exon 1F promoter methylation at pre-treatment was significantly associated with time since the first reported trauma, and at post-treatment with DRRI total life events (Figures 8A,B, respectively). There was no relationship between FKBP5 promoter methylation (at any time-point) with either the total number of negative life events or time since initial trauma.

Interestingly, DRRI total life events predicted PTSD symptom severity assessed by CAPS at post-treatment (*r* = 0.690, *n* = 16, *p* = 0.003). The three associations did not appreciably change when controlling for participant age at the time of the GR exon 1F promoter methylation assessment.

## Discussion

This is the first report in the literature to investigate cytosine methylation changes in association with changes in psychiatric symptoms and neuroendocrine measures in response to psychotherapy. In this small sample of responders and non-responders to PE psychotherapy, pre-treatment GR exon 1F promoter methylation predicted treatment outcome, but was not significantly altered in either group at post-treatment or follow-up. In contrast, pre-treatment cytosine methylation of the FKBP5 promoter did not predict treatment response, but decreased in association with recovery in veterans who no longer met diagnostic criteria for PTSD after psychotherapy. These findings distinguish two seemingly stable epigenetic markers that may associate, respectively, with prognosis (GR gene methylation) and symptom severity (FKBP5 gene methylation).

The focus in this study on the GR gene was based on observations that implicate enhanced GR sensitivity in PTSD (36). The focus on FKBP5, a co-chaperone of the GR cellular complex, is based on studies showing that FKBP5 inhibits the nuclear translocation of ligand-bound GR, thereby directly affecting functional GR sensitivity (12). FKBP5 gene expression is up-regulated by glucocorticoids through consensus glucocorticoid response elements (GREs) and by glucocorticoid-induced demethylation of the gene (12, 17, 52, 53). The underlying epigenetic mechanisms involved in the interaction of these two genes are not fully known, and remain of great interest. The findings of this study demonstrate distinct correlates with respect to PTSD for these two glucocorticoid related genes.

The lack of change over time in GR gene methylation is consistent with the idea that imprinting by early environmental experiences may result in enduring epigenetic changes in expression of this gene (1, 54). In animals, changes in GR gene methylation related to variations in maternal care are enduring, predicting GR responsiveness under a variety of experimental challenges in adulthood (5, 7, 55). Similarly, child maltreatment associates with hypermethylation of the GR exon 1F promoter in both post-mortem hippocampus (9) and leukocytes and an attenuated cortisol response to the Dex/CRH test in healthy adults (40). Our findings are consistent with the idea that environmental influences on the methylation of the GR exon 1F promoter are stable into adulthood and associated with clinical outcomes. Thus, methylation of this GR promoter was relatively stable across a 6-month period during which three independent measures were obtained under circumstances of changing symptom severity. This conclusion is buttressed by the strong inverse correlation between total number of negative life events as measured by the DRRI and GR gene 1F promoter methylation, as well as by the negative relationship between GR gene methylation and duration since initial trauma exposure. To our knowledge this finding is the first systematic documentation in humans of the stability of an epigenetic mark associated with childhood experience. In contrast, studies with rodents have been limited in most studies to a single assessment, typically in selected brain regions.

Variations in maternal care regulate hippocampal GR promoter methylation that, in turn, determines hippocampal GR expression, the efficiency of glucocorticoid negative-feedback regulation of hypothalamic CRF expression, and the magnitude of HPA-axis responses to stress (5, 56, 57). GR promoter methylation is thus an upstream regulator of GR gene expression and HPA-axis responsivity. In this study, GR promoter methylation at pre-treatment predicted HPA-axis activity following psychotherapy, but was not correlated with baseline cortisol measures at pre-treatment. Likewise in a recent cross-sectional study (40), GR promoter methylation in human lymphocytes did correlate with the cortisol response to the DEX/CRH challenge in a sample of healthy adults, many of whom had reported child abuse, but apparently not with basal measures of cortisol. It is possible, that cross-sectional correlations would have been observed herein following a corresponding level of HPA-axis manipulation as implicated by the DEX/CRF challenge. Nonetheless, the associations between GR exon IF promoter methylation and numerous functional glucocorticoid measures further increase confidence in the validity of the former to inform downstream processes related to functional neuroendocrine outcomes, but these associations may not necessarily be present when examined cross-sectionally.

Unlike GR promoter methylation, FKBP5 promoter methylation did not predict treatment response, but was correlated with measures of cortisol and glucocorticoid sensitivity. These findings are consistent with the role of FKBP5 as a moderator of intracellular GR signaling. These results are also consistent with our previous findings of an association between FKBP5 gene expression and plasma cortisol levels in WTC trauma survivors (21). The FKBP5 site in the current study differs from that examined by Klengel et al. (17), which associates with childhood adversity. This group examined regions (intronic regions and distal promoter region) of the FKBP5 gene that contain GREs. Methylation status in the intronic regions, especially of intron 7, mediated the effects of early life adversity on adult stress sensitivity since an association of child abuse with FKBP5 methylation at intron 7 has been reported, depending on FKBP5 genotype (17). Interestingly, methylation at these respective intronic regions in the rat, which also contain GREs, were decreased after a month of corticosterone administration resulting in an increase in FKBP5 gene expression strengthening the link between glucocorticoid levels and FKBP5 gene expression through epigenetic mechanisms that can also operate later in life (52, 53). Our choice of examining the proximal promoter region was prompted by the notion that methylation of this region would influence FKBP5 gene expression. Consistent with the findings of Klengel et al. (17), we found that increased levels of cortisol associated with decreased levels of FKBP5 promoter methylation.

**Figure 9:**
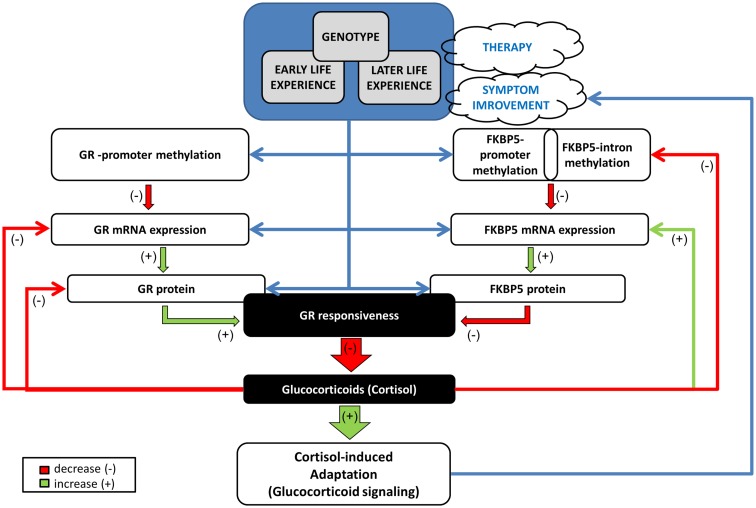
**Mechanistic model of the relationships/interactions between GR and FKBP5 methylation in PTSD**. Early life experience may impact both GR and FKBP5 gene methylation potentially in interaction with genotype. GR responsiveness is increased in PTSD, likely resulting from reduced GR methylation, with consequent increased GR expression, ultimately resulting in lowered cortisol levels. Low cortisol levels would serve to decrease FKBP5 gene expression through an intracellular loop mediated by GREs in the FKBP5 gene. Later life experience ± therapy, can also impact methylation of both genes, but most likely in distinct manners. A glucocorticoid-induced demethylation of FKBP5 will allow the subsequent increase in FKBP5 mRNA and protein expression, which would, in turn, ultimately decrease GR responsiveness, permitting the normalization of cortisol and of glucocorticoid signaling. This would have a beneficial effect on PTSD symptoms by impacting glucocorticoid responsive DNA sites that reduce sympathetic arousal or stimulate adaptation and recovery/resiliency (e.g., neuropeptide-Y). Green arrow denotes a positive influence (increase, “+”) and red arrow a negative influence (decrease, “−”). Blue arrow depicts a relationship. GR: glucocorticoid receptor encoded by the *NR3C1* gene, FKBP5: FK506 binding protein 5 encoded by the *FKBP51* gene.

These findings permit a distinction between biological markers associated with prognosis and treatment outcome. Thus, GR promoter methylation at pre-treatment was associated with treatment response, while dynamic variation in FKBP5 promoter methylation associated with treatment outcome. A model for understanding the unique relationships observed in GR and FKBP5 methylation and their potential interactions in PTSD is presented in Figure 9. Early experience may influence both GR and FKBP5 gene methylation. In PTSD, GR sensitivity is increased, likely resulting from reduced GR promoter methylation, which would ultimately result in lowered cortisol levels and, therefore, low glucocorticoid signaling. The low cortisol levels would serve to further decrease FKBP5 gene expression through an intracellular loop mediated by GREs in the FKBP5 gene. Decreased FKBP5 gene expression could serve to sustain an increased GR sensitivity. A decline in FKBP5 promoter methylation, such as occurred in treatment responders, might allow for an increase in FKBP5 gene expression, which would, in turn, ultimately decrease GR sensitivity. Thus, we found that treatment responders showed decreased FKBP5 promoter methylation, suggestive of increased FKBP5 gene expression, and measures of HPA-axis activity (i.e., plasma and urinary cortisol levels) reflecting decreased GR sensitivity. Likewise, higher levels of GR promoter methylation, suggestive of lower GR expression, were also associated with a positive response to treatment. Our previous studies suggest that increased GR sensitivity is a hallmark of PTSD (36). The mechanisms by which such dynamic changes in GR sensitivity associate with changes in PTSD symptoms remains to be fully elucidated; however the current findings suggest that the molecular mechanisms that regulate glucocorticoid signaling associate with treatment outcome.

In sum, this is the first demonstration of an epigenetic alteration in association with treatment response. This study represents an important initial step in establishing relevant molecular markers for PTSD therapies. In particular, the longitudinal approach in which symptoms vary over time is essential to distinguishing PTSD predictors from symptom correlates, and permits a more rational evaluation of potential treatment targets. The preliminary observations presented here require replication. Future prospective studies could detect the level of functional significance of small differences in methylation at baseline (as was the case for the GR-1F promoter) or small changes in methylation after an environmental challenge (as was noted for FKBP5 promoter methylation). However, the results support recent research indicating that some glucocorticoid related genes may be subject to environmental regulation throughout life (58). Moreover, the data suggest that psychotherapy resulting in substantial symptom change constitutes a form of “environmental regulation” that may alter epigenetic state. Finally, the results demonstrate that different genes may be associated with prognosis and symptom state, respectively.

## Conflict of Interest Statement

The authors declare that the research was conducted in the absence of any commercial or financial relationships that could be construed as a potential conflict of interest.
